# Replicative transposition contributes to the evolution and dissemination of KPC-2-producing plasmid in *Enterobacterales*

**DOI:** 10.1080/22221751.2021.2013105

**Published:** 2021-12-21

**Authors:** Yu Tang, Gang Li, Pinghua Shen, Ying Zhang, Xiaofei Jiang

**Affiliations:** a Department of Laboratory Medicine, Huashan Hospital, Shanghai Medical College, Fudan University, Shanghai, People’s Republic of China; b Department of Laboratory Medicine, Shanghai East Hospital, Tongji University School of Medicine, Shanghai, People’s Republic of China; c Department of Laboratory Medicine, Jinshan Hospital, Shanghai Medical College, Fudan University, Shanghai, People’s Republic of China; d Department of Laboratory Medicine, Shanghai First Maternity and Infant Hospital, Tongji University School of Medicine, Shanghai, People’s Republic of China; e Department of Laboratory Medicine, Obstetrics and Gynecology Hospital, Fudan University, Shanghai, People’s Republic of China

**Keywords:** *Enterobacterales*, *bla*_KPC-2_, replicative transposition, evolution, dissemination

## Abstract

*Klebsiella pneumoniae* carbapenemase (KPC)-producing *Enterobacterales* are prevalent worldwide and pose an alarming threat to public health. The incidence and transmission of *bla*_KPC-2_ gene via horizontal gene transfer (e.g. transposition) have been well documented. However, the dynamics of transposon structure bearing *bla*_KPC-2_ and their exact effects on the evolution and dissemination of *bla*_KPC-2_ gene are not well characterized. Here, we collected all 161 carbapenem-resistant *Enterobacterales* (CRE) isolates during the early stage of CRE pandemic. We observed that the prevalence of KPC-2-producing *Enterobacterales* was mediated by multiple species and sequence types (STs), and that *bla*_KPC-2_ gene was located on three diverse variants of Tn*1721* in multi-drug resistance (MDR) region of plasmid. Notably, the outbreak of KPC-2-producing plasmid is correlated with the dynamics of transposon structure. Furthermore, we experimentally demonstrated that replicative transposition of Tn*1721* and IS*26* promotes horizontal transfer of *bla*_KPC-2_ and the evolution of KPC-2-producing plasmid. The Tn*1721* variants appearing concurrently with the peak of an epidemic (A2- and B-type) showed higher transposition frequencies and a certain superior ability to propagation. Overall, our work suggests replicative transposition contributes to the evolution and transmission of KPC-2-producing plasmid and highlights its important role in the inter- and intra-species dissemination of *bla*_KPC-2_ gene in *Enterobacterales*.

## Introduction

*Klebsiella pneumoniae* carbapenemase (KPC) is a class A serine *β*-lactamase that efficiently hydrolyzes most *β*-lactam antimicrobial agents, including carbapenems, limiting treatment options in infected patients seriously [1]. KPC-producing *Enterobacterales*, particularly *K. pneumoniae*, have spread worldwide over the last decade, becoming an urgent public health threat [2]. KPC-2, the most commonly identified variant, is a dominant factor leading to carbapenem resistance in *Enterobacterales*. The *bla*_KPC-2_ gene is typically identified in mobile transposon, which is most often situated on conjugative plasmids [2–4]. Tn*4401* is the major vehicle of *bla*_KPC-2_ in most countries and regions, such as Europe [5], the United States [6], and Brazil [7]. In Asia, *bla*_KPC-2_ is mostly located on diverse variants of Tn*1721* and IS*26* [8–10].

Horizontal gene transfer (HGT) plays an important role in the evolution of bacteria and the dissemination of antibiotic resistance genes [11]. Tn*4401* and Tn*1721*, typical replicative transposons belonging to Tn*3* family, have been proved to mobilize *bla*_KPC-2_ at a high transposition frequency, and the latter is capable of transferring *bla*_KPC-2_ both internal and external to this element [12,13]. However, few published studies provided comprehensively analysis of epidemic of KPC-2-producing *Enterobacterales*, the dynamics of genetic structure surrounding *bla*_KPC-2_ and transposition mechanism of these elements. Notably, *bla*_KPC-2_ is located on diverse variants of Tn*1721* that exhibited various transposition frequencies and movement patterns [13], and the mechanism of movement of A2-type (Tn*1721*-*bla*_KPC-2_-IS*26*) remains undetermined.

Here, we show that outbreak of KPC-2-producing plasmid is correlated with the dynamics of transposon structure in *Enterobacterales*. The A2- and B-type Tn*1721* appearing concurrently with the peak of the epidemic of *bla*_KPC-2_-carrying isolates demonstrated higher transposition frequency. Their specific replicative transposition (the IS*26* pattern for A2-type and the Tn*1721-bla*_KPC-2_-IRL2 pattern for B-type) had a certain superior ability to conjugate into another strain. Thus, replicative transposition contributes to the evolution and dissemination of KPC-2-producing plasmid in *Enterobacterales*, facilitating the inter- and intra-species dissemination of *bla*_KPC-2_.

## Materials and methods

### Media and growth of strains

Bacteria were routinely cultured at 37°C in Luria–Bertani (LB) medium or on LB agar. Plasmids were constructed in *Escherichia coli* DH5α (*supE44 Δ lacU169 [ϕ 80 lacZ ΔM15] hsdR17 recA1 endA1 gyrA96 thi-1 relA1*). HB101 (*recA13* F^-^ STR^r^) and *E. coli* J53 (AZ^r^) were used as recipient strains in transposition experiments and conjugation experiments, respectively. The following antibiotics were added at the indicated concentrations: imipenem (IPM), 1 mg/L; streptomycin (STR), 25 mg/L; trimethoprim (TMP), 25 mg/L and sodium azide (AZ), 100 mg/L.

### Clinical isolates

A total of 161 non-duplicated, carbapenem-resistant *Enterobacterales* (CRE) isolates were collected from August 2006 to December 2010 during routine identification and antimicrobial susceptibility testing by the Microbiology Laboratory, Huashan Hospital, Fudan University (Shanghai, China). This collection comprised all clinical isolates from the first occurrence of KPC-2-producing isolates (*K. pneumoniae*) to the prevalence of this cabapenemase (Dataset S1). For comparison, a susceptible collection of 112 carbapenem-sensitive *K. pneumoniae* (CS-KP) were also collected contemporaneously from similar departments (Dataset S2).

### Multilocus sequence typing (MLST) and pulsed-field gel electrophoresis (PFGE)

MLST was performed according to the protocol described on the Pasteur Institute MLST website (https://bigsdb.pasteur.fr/klebsiella/klebsiella.html for *K. pneumoniae* and https://bigsdb.pasteur.fr/ecoli/ecoli.html for *E.coli*) and PubMLST website (https://pubmlst.org/organisms/citrobacter-spp for *Citrobacter freundii* and https://pubmlst.org/organisms/klebsiella-aerogenes for *Klebsiella aerogenes*). Genetic relatedness among the ST11 *K. pneumoniae* isolates was analyzed by XbaI-PFGE type as described previously [9]. Dendrograms were conducted using the Dice coefficient and the unweighted pair-group method using average linkage clustering [14].

### PCR screening of *bla*_KPC-2_ and IncFII replicon and analysis of genetic environment of *bla*_KPC-2_ gene among clinical isolates

The *bla*_KPC-2_ were identified through the amplification and sequence analysis of a 750-bp polymerase chain reaction (PCR) product [15]. IncFII replicon screen was conducted by PCR-based replicon typing using previously reported primers [16]. The *bla*_KPC-2_-bearing genetic structures were determined by a series of PCR assays as reported previously [15].

### Transformation and conjugation experiments of *bla*_KPC-2_-bearing plasmids

Thirty-five *bla*_KPC-2_-bearing plasmids were obtained by transformation or conjugation (Dataset S3). Plasmids of clinical isolates were extracted with a Qiagen Plasmid Midi kit (Qiagen, Germany) and examined by agarose gel electrophoresis, and then transformed into *E. coli* DH5α Electrocompetent cells by electroporation (Micro-Pulser electroporator; Bio-Rad, USA). Conjugation experiment was performed with *E. coli* J53 (AZ^r^) as the recipient. Transformants and conjugants were selected on MacConkey agar containing IPM (AZ was also used in conjugants selection) and identified by VITEK 2 system (bioMérieux, France) and were further subjected to PCR amplification of IncFII replicons, *bla*_KPC-2_ and genetic environment of *bla*_KPC-2_ according to our previous operations. For plasmids originated from clinical isolates of *E. coli*, each transformant was also identified by detecting the deletion of *lacY* gene as *E. coli* DH5α lacks the *lac* operon. Primers are listed in Table S1.

### Bioinformatics analysis

All complete genome sequences of *Enterobacterales* publicly available (5152 in total) and all of the plasmid sequences harbored in these strains (10,507 in total) were downloaded from NCBI database in August 2021 (Chromosomes in Dataset S4 and plasmids in Dataset S5). The *bla*_KPC-2_ gene was identified by using nucleotide BLAST. Plasmid incompatibility type was determined by comparing with information in the Plasmid MLST locus/sequence definitions database (https://pubmlst.org/bigsdb?db=pubmlst_plasmid_seqdef& page = sequenceQuery).

### Plasmids construction

The primers and plasmids used in this study are listed in Table S1 and Table S2, respectively. pHS10842 (GenBank accession no. KP125892), a vector favorable for use in the exploration of the transposition mechanism of Tn*1721*-like transposons, has been described previously [13].

For pHS10842-*ΔtnpA*_Tn*1721*_, IRR and *tnpR* fragments were amplified with primers JP934/JP935 and JP936/JP937, respectively. The IRR fragment share 20-bp sequences with AhdI restriction sites of pHS10842 and the *tnpR* fragments, respectively. The *tnpR* fragments also share 20-bp sequences with the IRR fragment and AflII restriction sites of pHS10842, respectively. The two fragments were subcloned into AhdI and AflII restriction sites of pHS10842 by use of the NEBuilder HiFi DNA assembly master mix (New England BioLabs, USA), generating the derivative with the *tnpA* deletion of Tn*1721*, pHS10842-*ΔtnpA*_Tn*1721*_.

The transposase deletion was introduced into *IS26* by digesting plasmid pHS10842 with SwaI and XmnI to removal a 560-bp fragment (base 11,366 to base 11,925 in GenBank accession no. KP125892) and generate blunt ends. The DNA was ligated and transformed into competent cells of *E. coli* DH5α. pHS10842-*ΔtnpA*_Tn*1721*_*Δtnp26* was constructed in a similar strategy with pHS10842-*ΔtnpA*_Tn*1721*_.

### Transposition assays and molecular characterization of transposition events

Transposition assays were performed as described previously [13,17]. Transposition frequency is calculated as the number of IPM^r^ TMP^r^ STR^r^ transconjugants per TMP^r^ STR^r^ transconjugant. For each transposition event, the movement patterns were determined by agarose gel electrophoresis and Southern hybridization [13]. The exact insertion site and target site duplication of Tn*1721* and IS*26* were determined by using primes specific for regions internal to Tn*1721* and IS*26* and primers specific for R388 as described previously [13]. Primers are listed in Table S1.

### Analysis of the target site consensus sequence

The relative frequencies of the AT and GC contents of the region extending from 50 bp upstream to 50 bp downstream of the duplicated target site for IS*26* (8 bp) were calculated and plotted on a line graph. The pictures of the relative frequencies of the bases at each position were generated with the Pictogram program (http://genes.mit.edu/pictogram.html).

### Statistics

Statistical significance was assessed by Fisher’s exact test or Chi-square test with Yates’ correction using GraphPad Prism8 software (https://www.graphpad.com/). *P *< 0.05 was considered statistically significant.

## Results

### The prevalence of KPC-2-producing *Enterobacterales* was mediated by multiple species and STs

**Figure 1.**
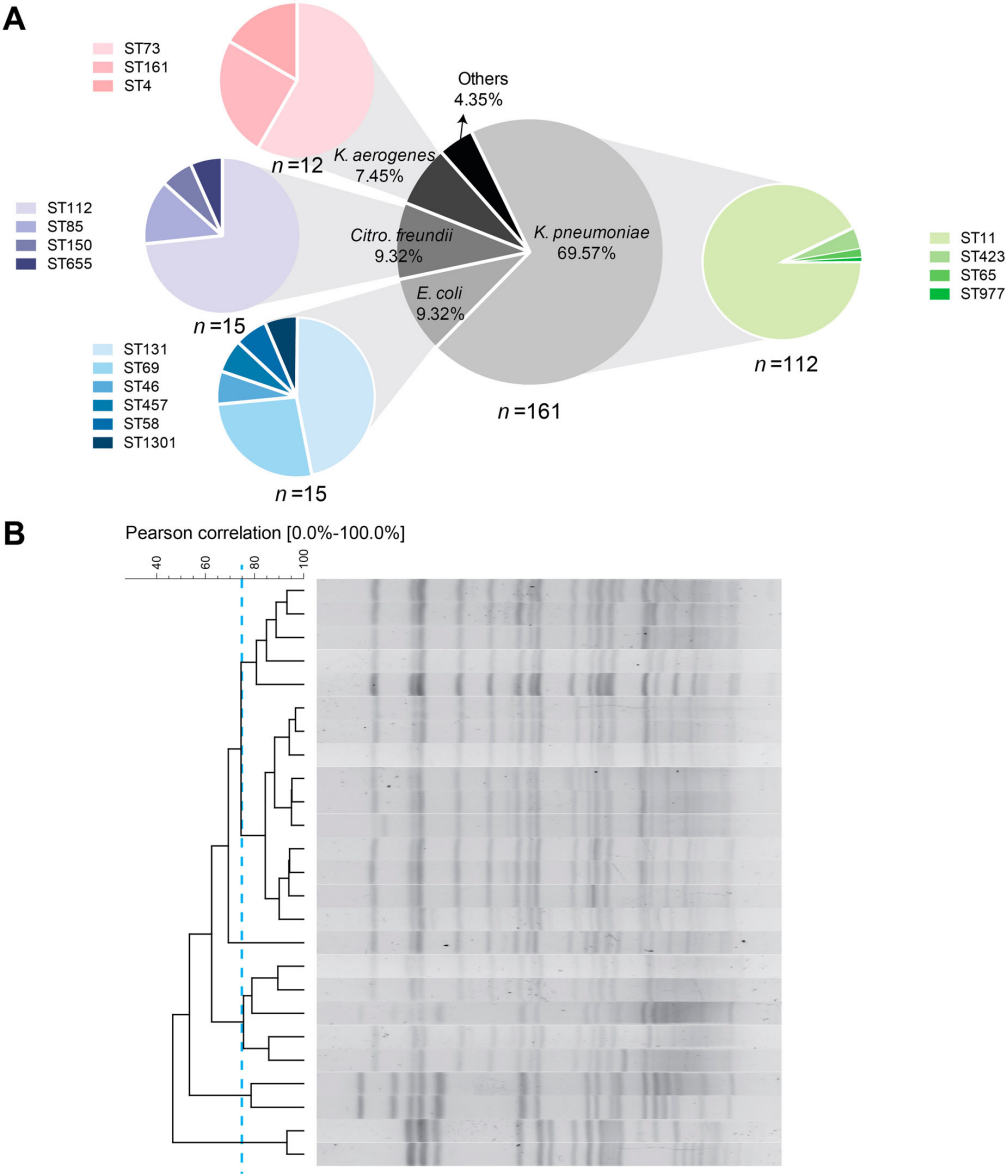
The molecular epidemiological investigation of CRE isolates (A) Species distribution and sequence types (STs) of *K. pneumoniae, E. coli, Citro. freundii* and *K. aerogenes*. (B) Dendrogram of ST11 *K. pneumoniae* isolates based on pulsed-field gel electrophoresis (PFGE) of XbaI-digested DNA.

All of the 161 CRE were identified as *bla*_KPC-2_-positive, including *K. pneumoniae* (112, 69.56%), *E. coli* (15, 9.32%), *Citro. freundii* (15, 9.32%), *K. aerogenes* (12, 7.45%) and other species (7, 4.35%) (Figure 1A). Among the 112 *K. pneumoniae* isolates, ST11 was the most prevalent ST, followed by ST423, ST65 and ST977, and PFGE of ST11 *K. pneumoniae* indicated five diverse subtypes with a criterion of 75% identity (Figure 1B). For comparison, we collected CS-KP as susceptible controls that were matched by time and ward. As expected, all CS-KP isolates were *bla*_KPC-2_-negative and the STs of the susceptible collection were scattered without any dominant STs (Figure S1). *E. coli, Citro. freundii* and *K. aerogenes* isolates were comprised of several STs. Together, these results suggested that *bla*_KPC-2_ is the chief culprit leading to carbapenem resistance and that the prevalence of *bla*_KPC-2_ in *Enterobacterales* was mediated by multiple species and STs, rather than clonal spread.

### *bla*_KPC-2_ genes were usually located on three diverse variants of Tn*1721* in MDR region of plasmid

The *bla*_KPC-2_ genes were usually reported to be located on MDR region of plasmid, and IncFII plasmids contributed significantly to the global prevalence of *bla*_KPC_ among *K. pneumoniae* [4,9]. To determine the correlation between IncFII plasmids and *bla*_KPC-2_ gene, we conducted a bioinformatic analysis of 5152 complete genome sequences of *Enterobacterales* publicly available (including 5152 chromosomes and 10,506 plasmids in total, Dataset S4 and Dataset S5). Among these, 311 strains were identified to be *bla*_KPC-2_-positive, and the overwhelming majority of *bla*_KPC-2_ genes were found on plasmids (300/311, 96.46%). Remarkably, most of the *bla*_KPC-2_-bearing plasmids belonged to IncFII group, but this group was less common in *bla*_KPC-2_-negative plasmids [174/300 (58%) versus 2195/10206 (21.51%), *P *< 0.0001; Table 1]. Moreover, IncFII plasmids were significantly over-represented in *bla*_KPC-2_-positive *K. pneumoniae* compared with that in *bla*_KPC-2_-negative group [151/204 (74.02%) versus 657/2701 (24.32%), *P *<* *0.0001; Table 1]. These findings suggest that *bla*_KPC-2_ genes are mostly located on plasmids in *Enterobacterales*, and that IncFII is the most common Incompatibility group, especially in *K. pneumoniae*.

**Table 1.** Incompatibility group analysis of plasmids with or without *bla*_KPC-2_ obtained from NCBI database.

Plasmid host	*bla*_KPC-2_-positive^a^	*bla*_KPC-2_-negative	*P-*value
*K. pneumoniae*	151/204 (74.02%)	657/2701 (24.32%)	< 0.0001
*E. coli*	5/24 (20.83%)	780/3716 (20.99%)	0.8161
*Citro. freundii*	3/20 (15%)	42/274 (15.33%)	1.0000
*K. aerogenes*	1/3 (33.33%)	1/48 (2.08%)	0.1153
Others	14/49 (28.57%)	715/3467 (20.62%)	0.2359
In total	174/300 (58%)	2195/10206 (21.51%)	< 0.0001

^a^ Data are number of IncFII plasmids/total number (% of IncFII-positive rates). *P-*value for comparisons of the IncFII-positive rates of *bla*_KPC-2_-positive and *bla*_KPC-2_-negative groups.

**Figure 2.**
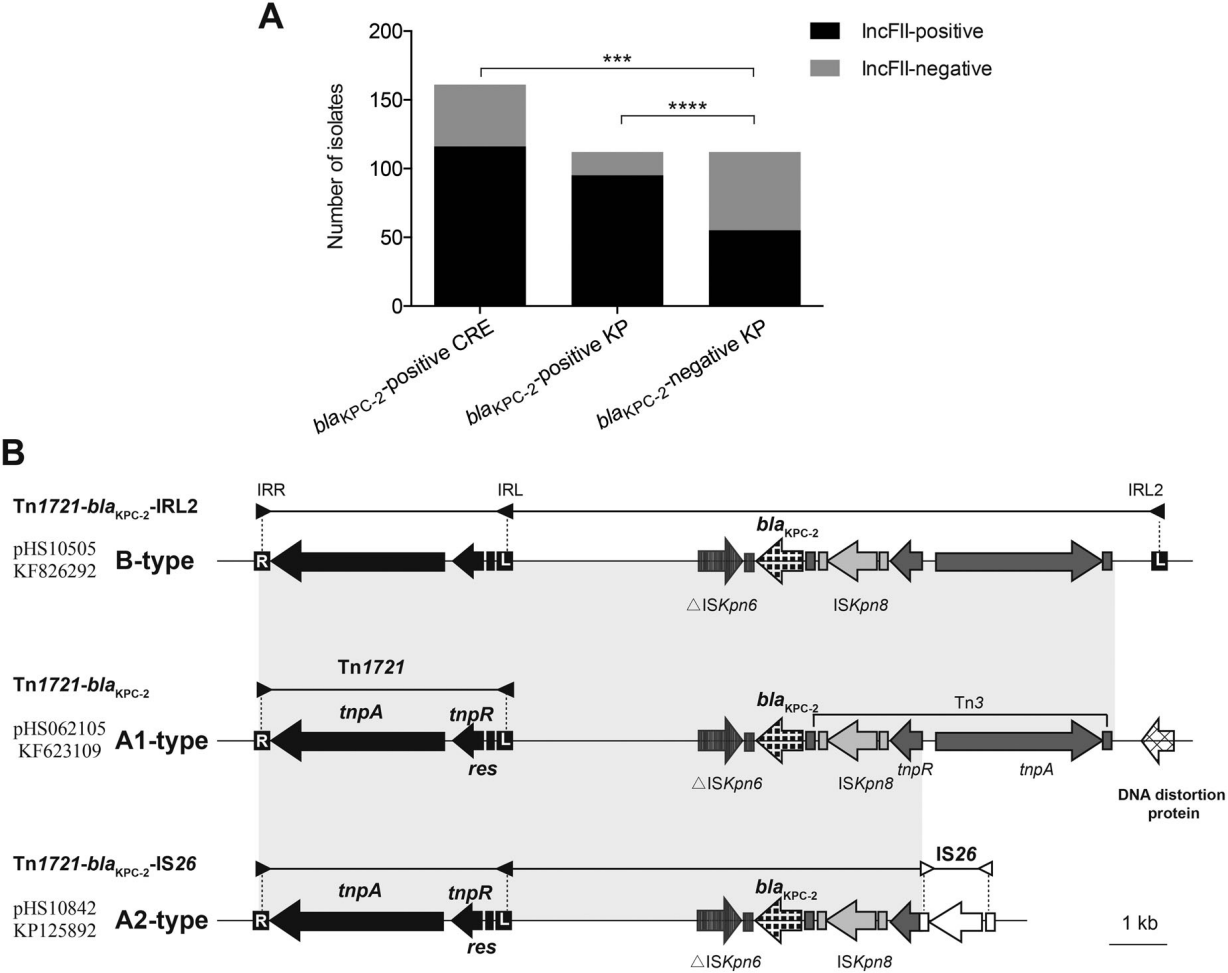
IncFII screen of clinical isolates and genetic structures surrounding *bla*_KPC-2_. (A) IncFII-positive rates of *bla*_KPC-2_-positive and *bla*_KPC-2_-negative groups. (B) Schematic representation of *bla*_KPC-2_-bearing genetic elements classified as A1-, A2- and B-type. Genes are depicted as arrows according to the direction of transcription. *bla*_KPC-2_ is shown as spotted arrows. Inverted repeats are indicated by rectangles in different colors: Tn*1721* (black), Tn*3* (dark gray), IS_Kpn8_ (light gray), and IS*26* (white). Regions sharing identical sequences across plasmids are indicated by gray shading between the different representations of the plasmids. The GenBank accession numbers for pHS10505, pHS062105, and pHS10842 are KF826292, KF623109, and KP125892, respectively.

To evaluate this correlation in clinical isolates, we first performed IncFII replicon screening on all clinical isolates. Significantly, the detection rate of IncFII replicon was 84.82% in *K. pneumoniae* and the rate was slightly lower for *E. coli* (73.33%) and *K. aerogenes* (66.67%) (Table S3). None of *Citro. freundii* isolates was detected to IncFII-positive. However, the rate was much lower in carbapenem-susceptible collection in comparison with either CR-KP or CRE collection (49.11% vs. 84.82% for CR-KP, *P *<* *0.0001; 49.11% vs. 72.05% for CRE, *P *=* *0.0001, Figure 2A). Next, the linkage between IncFII plasmids and *bla*_KPC-2_ gene was determined in thirty-five transformants and conjugants containing *bla*_KPC-2_-positive plasmid by PCR amplification of IncFII replicons and genetic environment of *bla*_KPC-2_ (see Dataset S3 for details). Interestingly, 80% of plasmids carrying *bla*_KPC-2_ gene (28/35) belong to IncFII group. Finally, five plasmids in various sizes (two IncFII-negative and four IncFII-positive, Dataset S3) were selected for complete sequence analysis to furtherly confirm the correlation between IncFII plasmids and *bla*_KPC-2_ gene.

Moreover, our analysis of genetic environment of *bla*_KPC-2_ gene among 161 CRE identified three diverse variants of Tn*1721* bearing *bla*_KPC-2_ (designated A1-, A2- and B-type) (Figure 2B). All of them possess a core element (Tn*1721*) that consists of two 38-bp terminal inverted repeats (IRs; the right inverted repeat [IRR] and the left inverted repeat [IRL]), transposase (TnpA), resolvase (TnpR) and resolution site (*res*). The B-type is composed of Tn*1721*, *bla*_KPC-2_, an additional inverted repeat (IRL2), as well as several unrelated elements (Tn*3*, IS*Kpn6*, and IS*Kpn8*). The sequence of A1-type (Tn*1721*-*bla*_KPC-2_) shares 100% identity with that of B-type, but it does not contain the additional IRL, IRL2. In the A2-type, Tn*3* was disrupted by an IS*26*, forming the chimera, Tn*1721*-*bla*_KPC-2_-IS*26*. The A2-type Tn*1721* accounted for more than half the samples (59.01%, 95/161), and A1- and B-type accounted for 19.25% (31/161) and 18.63% (30/161), respectively (Table 2 and Figure 2B).

**Table 2.** Detection rate of Tn*1721*-like transposons bearing *bla*_KPC-2_ on 161 CRE.

Tn*1721* types	Genetic structure surrounding *bla*_KPC-2_	Detection of *K. pneumoniae*	Detection of other *Enterobacterales*	Detection of *Enterobacterales* (%)
A1	Tn*1721*-*bla*_KPC-2_	10	21	31 (19.25%)
A2	Tn*1721*-*bla*_KPC-2_-IS*26*	73	22	95 (59.01%)
B	Tn*1721*-*bla*_KPC-2_-IRL2	28	2	30 (18.63%)
Others	Undefined	1	4	5 (3.11%)

### Outbreak of KPC-2-producing plasmids is correlated with the dynamics of transposon structure

**Figure 3.**
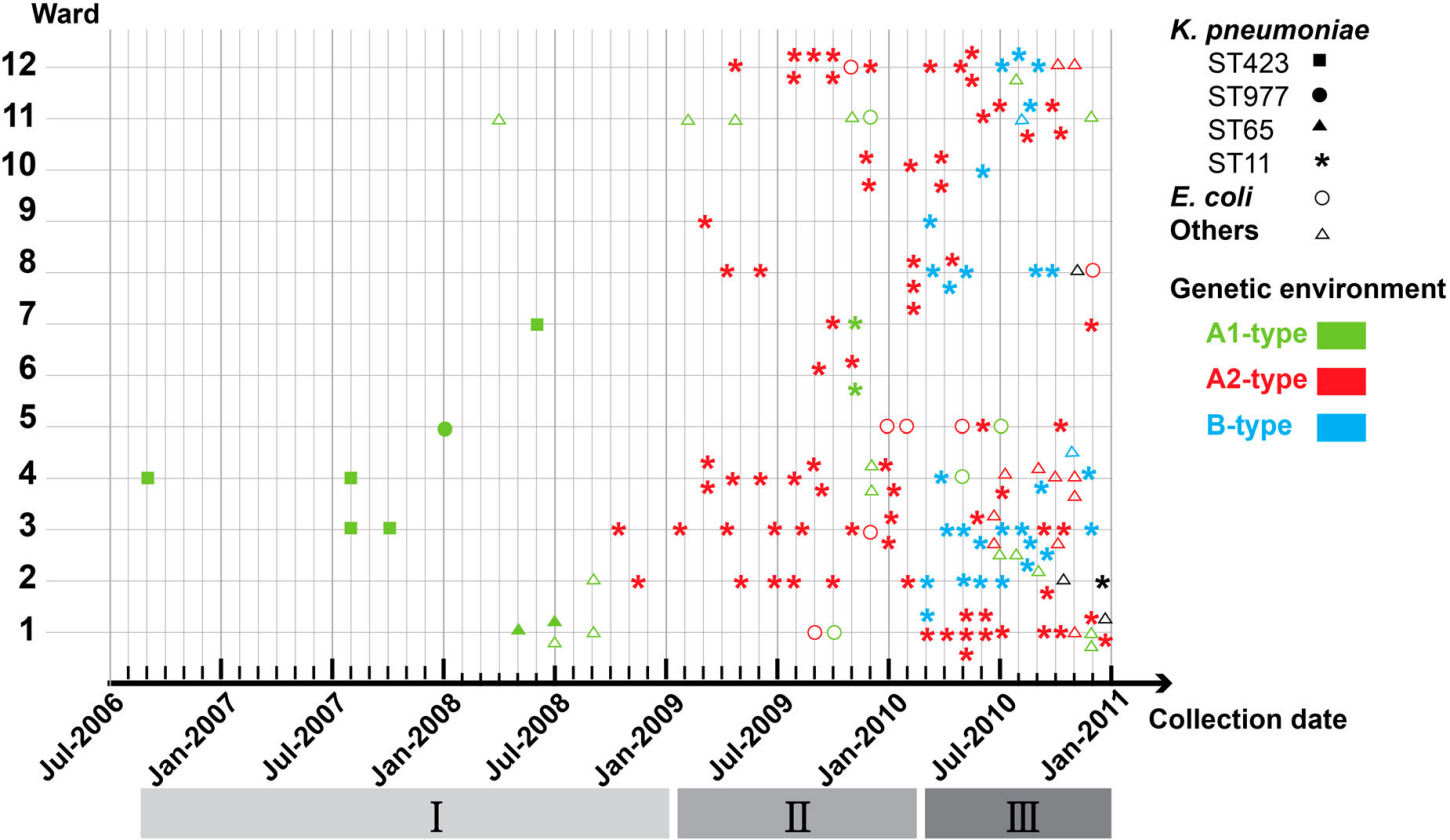
Overview of 161 CRE clinical isolates collected from Huashan Hospital. *K. pneumoniae* isolates are shown as solid signs: squares (ST423), circles (ST977), triangles (ST65), and asterisks (ST11). Other *Enterobacterales* isolates are shown as empty signs: circles (*E. coli*) and triangles (others). Isolates are plotted based on ward (vertical axis) and time of collection (horizontal axis). Color indicates distinct Tn*1721* variants carrying *bla*_KPC-2_: A1-type (green), A2-type (red), B-type (blue) and undefined (black).

In order to clarify the progression of *bla*_KPC-2_ outbreak, we comprehensively analyzed all data obtained in molecular epidemiological investigation. Figure 3 provides an overview of the evolution of *bla*_KPC-2_ bearing structure in *Enterobacterales*. Samples are plotted by month of isolation, wards, species, STs of most common resistant specie (*K. pneumoniae*) and transposable elements carrying *bla*_KPC-2_ gene. As shown in Figure 3, *bla*_KPC-2_ genes that were located in three distinct Tn*1721*-like transposons on plasmid, originated from *K. pneumoniae*, then became increasingly prevalent in this specie and spread in *Enterobacterales* further. The epidemic of *bla*_KPC-2_-carrying plasmid reported here had three stages, designated as (i) distributed period (August 2006–April 2009, 19 isolates), (ii) epidemic period (May 2009–February 2010, 50 isolates), and (iii) mixed epidemic period (March–December 2010, 92 isolates). In the distributed period, *bla*_KPC-2_ appeared sporadically in *K. pneumoniae* without any predominate STs, and all Tn*1721*-like transposons belonged to A1-type. At the second epidemic stage, KPC-2-producing plasmid were prevalent in *K. pneumoniae* ST11 that consisted of several different subtypes, and spread into other *Enterobacterales*. A2-type Tn*1721* was the dominant structure carrying *bla*_KPC-2_. In the mixed epidemic period, KPC-2-producing isolates increased furtherly. This stage involved three distinct Tn*1721* variants, and the number of emerging B-type was almost equal to that for A2-type during the same period.

Altogether, outbreak of KPC-2-producing plasmid was correlated with the dynamics of *bla*_KPC-2_-bearing transposon structure, and A2- and B-type Tn*1721* appeared concurrently with the peak of *bla*_KPC-2_ epidemic.

### Replicative transposition promotes horizontal transfer of bla_KPC-2_

A critical step in the dissemination process of *bla*_KPC-2_ is the HGT of mobile genetic elements (MGEs) surrounding this determinant. According to our comprehensive analysis of epidemiological data, it was presumed that replicative transposons, Tn*1721* and IS*26,* can mobilize *bla*_KPC-2_ through transposition, promoting the dissemination of *bla*_KPC-2_ in *Enterobacterales*. In our previous study, A1- and B-type Tn*1721* have been shown to transfer *bla*_KPC-2_ both internal and external to this element, and target transposition into 5-bp region that gradually exhibits a degenerated degree of AT-rich regions from both sides to the middle and that is immediately flanked by GC-rich regions [13]. Here, we characterized the movement and target site of A2-type Tn*1721* (Tn*1721*-*bla*_KPC-2_-IS*26*).

**Figure 4.**
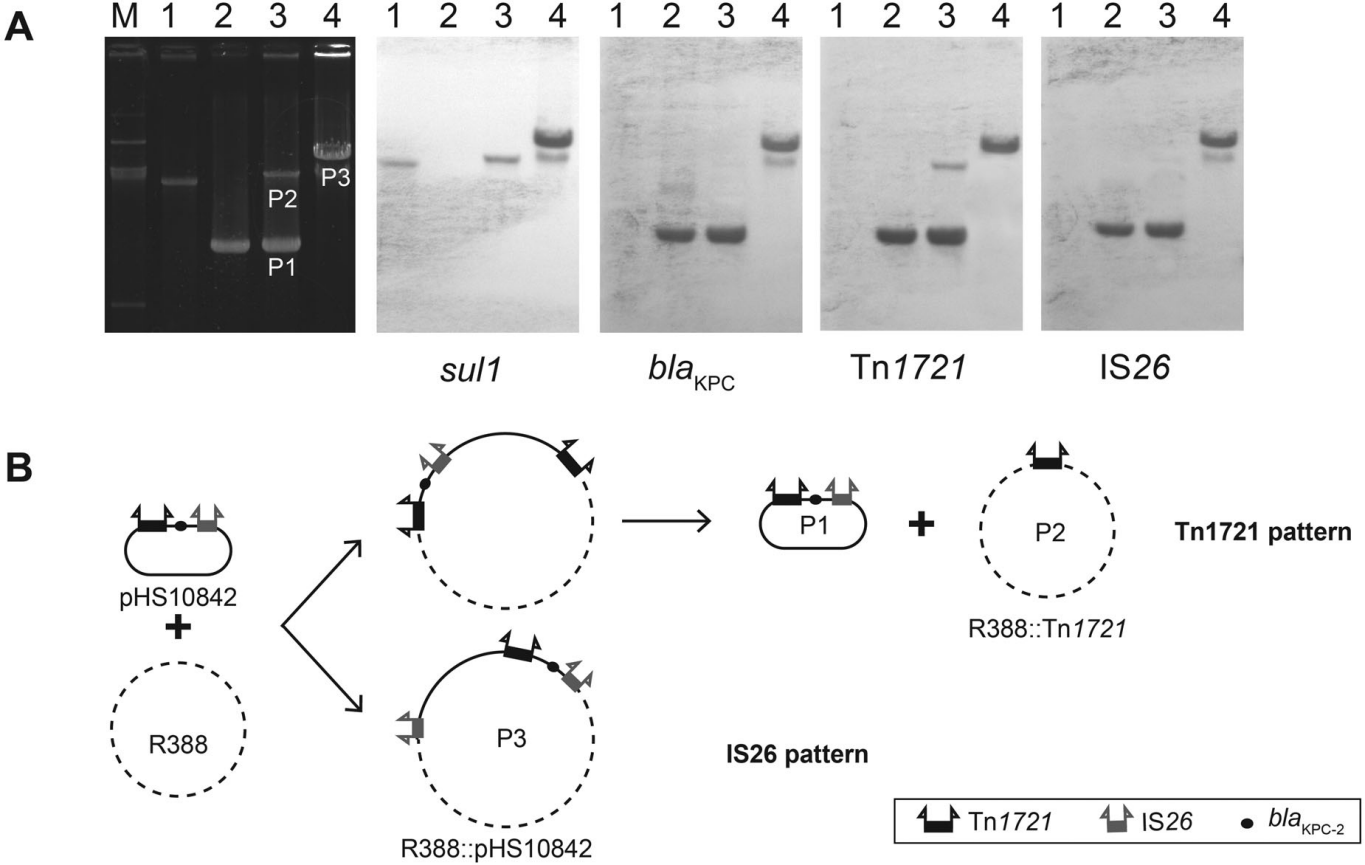
Transposition patterns in A2-type (Tn*1721*-*bla*_KPC-2_-IS*26*). (A) Southern hybridization of plasmids in typical transconjugants. The electrophoretic profiles of the plasmids and hybridization with the *sul1*-specific probes (*sul1* is a genetic marker of R388), *bla*_KPC-2_-specific probes, Tn*1721*-specific probes, and IS*26*-specific probes are shown. (B) Two transposition patterns (the Tn*1721* pattern and the IS26 pattern) in Tn*1721*-*bla*_KPC-2_-IS*26* structure.

Two distinct patterns of movement mediated by Tn*1721* and IS*26* existed in this chimera. Tn*1721* pattern was the same as the one detected in A1- and B-type Tn*1721* previously, including cointegrate forming and resolving steps [13]. In addition to having the Tn*1721* pattern, a different IS*26* pattern via replicative transposition was also detected in several cases of A2-type Tn*1721* (Figure 4B). Only one plasmid (P3) was obtained from the transconjugant. The donor (pHS10842) and target (R388) plasmids generated this cointegrate (P3) in which both plasmids fused together by directly repeated copies of IS*26*. This result was supported by a series of data (Figure 4A), as follows. (i) the size of P3 was larger than that of R388 and P2. (ii) Southern blot analysis showed that P3 contained *bla*_KPC-2_, Tn*1721*, *sul1*, IS*26*. (iii) A series of PCRs confirmed that P3 had the same *bla*_KPC-2_-bearing genetic structure (Tn*1721*-*bla*_KPC-2_-IS*26*) as that of pHS10842, and the junctions between donor and target in each case were amplified and sequenced with primers specific for regions internal to IS*26* and primers specific for R388.

**Figure 5.**
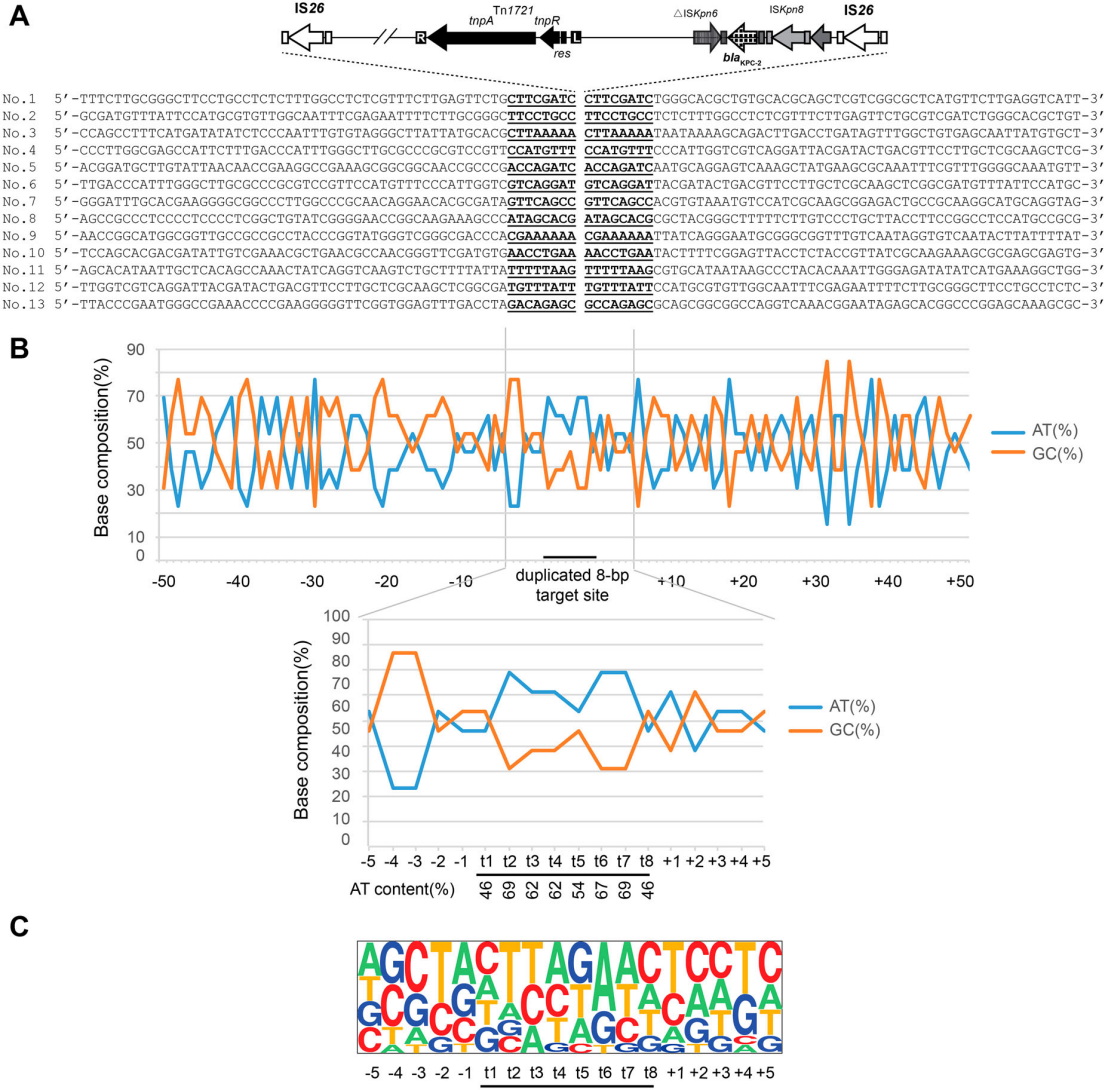
Target site analysis of IS*26*. (A) Sequence alignment of 13 transposition events of IS*26* in R388 plasmid. The duplicated 8-bp target site sequence is underlined. The 50 nucleotides upstream and downstream of the target sites are shown. (B) Statistical analysis of the nucleotides at the 13 transposition sites. The percentages of AT and GC residues at each position from 50 nucleotides upstream to 50 nucleotides downstream of the target site are shown. The 8-bp duplication of target site, here named t1, t2, t3, t4, t5, t6, t7, and t8 are designated by a black bar. The percentage of AT and GC residues in the region spanning positions −5 to 5 are also indicated in the lower graphs. (C) Pictogram showing the relative frequencies of each A, T, C, and G residue at the target site. The data were deduced from the 13 experimental transposition events shown in panel A.

Consistent with previous reports [18], 8-bp target site duplication was evidenced for each transposition events (Figure 5). At the target sites, the AT content for regions from t2 to t7 stabilize at 60-70%, while the AT content at t1 and t8 was slightly lower (46%). Nucleotide composition analysis revealed the t2 and t3 positions were found to be predominantly T residues (54% and 38%, respectively), while t6 and t7 positions were mostly A residues with the same percentage as that for t2 and t3, respectively. It is noteworthy that the insertion sites mostly carried one or even more AA or/and TT nucleotide tandems. These data suggested that IS*26* preferentially targets AT-rich regions with AA and TT nucleotide tandems.

The transposition frequency of A2-type Tn*1721* was measured to be 3.8 × 10^−6^ (Table 3). This represented a 4-fold increase in efficiency compared to that of A1-type, a *bla*_KPC-2_-bearing structure lacking IS*26*, and 30% of that for B-type Tn*1721* containing an additional left inverted repeat (IRL2). The *tnpA* deletion of either Tn*1721* or IS*26* decreased half of the frequency, and both *tnpA* deletions led to a functional inability to mobilize *bla*_KPC-2_. Hence, *bla*_KPC-2_ embedded between Tn*1721* and IS*26* was transferred at an apparently higher frequency owing to the existence of both elements.

**Table 3.** Transposition frequency of Tn*1721*-like transposons and derivatives^a^.

Donor plasmid	No. of independent determinations	Transposition frequency^b^	
		Mean	Range
pHS10842-Tn*1721*B	5	1.13 × 10^−5^	8.15 × 10^−6^–16 × 10^−5^
pHS10842-Tn*1721*A1	4	0.95 × 10^−6^	0.4 × 10^−6^–1.57 × 10^−6^
pHS10842 (A2)	4	3.8 × 10^−6^	3.4 × 10^−6^–4.2 × 10^−6^
pHS10842-*ΔtnpA*_Tn*1721*_	4	1.7 × 10^−6^	1.1 × 10^−6^–1.9 × 10^−6^
pHS10842-*Δtnp26*	4	1.3 × 10^−6^	1.0 × 10^−6^–1.9 × 10^−6^
pHS10842-*ΔtnpA*_Tn*1721*_*Δtnp26*	4	< 9 × 10^−8^	< 8.3 × 10^−8^–< 1.0 × 10^−7^

^a^ In all cases, the target was R388.

^b^ Transposition frequency is expressed as the number of IPM^r^ TMP^r^ STR^r^ transconjugants per TMP^r^ STR^r^ transconjugant.

Taken together, our findings indicate that all three transposons were capable of transferring *bla*_KPC-2_ through replicative transposition, and that A2- and B-type Tn*1721* showed higher transposition frequencies and had a certain superior ability to propagation.

### Replicative transposition of Tn1721 contributes to the evolution and dissemination of KPC-2-producing plasmid

Given that Tn*1721* variants have been shown various capacities of transferring *bla*_KPC-2_ via replicative transposition, an attractive hypothesis is that the A2- and B-type Tn*1721* occasionally appearing in the late-stage stood out from the rest through selection for their ability to facilitate dissemination of *bla*_KPC-2_. As discussed above, the outbreak of KPC-2-producing plasmid was correlated with the dynamics of *bla*_KPC-2_-bearing Tn*1721* structure, and A2- and B-type Tn*1721* arising concurrently with the peak of *bla*_KPC-2_ epidemic showed apparently higher transposition frequencies. Such an association and different movement capabilities imply that replicative transposition of Tn*1721* variants contributes to the evolution and dissemination of KPC-2-producing plasmid in *Enterobacterales.* This mechanism could enable *bla*_KPC-2_ gene to search for suitable host spontaneously, dealing with the antibiotic pressure from the environment.

## Discussions

KPC-producing *Enterobacterales* that have spread extensively throughout the world, are an important cause of nosocomial infections, especially urinary tract infections, respiratory tract infections, and bloodstream-associated infections [1]. During the last decade, several studies have described of diverse transposable elements surrounding *bla*_KPC-2_ gene [9,10,12]. However, little is known about the exact impacts of these genetic environment change and its correlation with epidemic of *bla*_KPC-2_ gene. Our investigation of clinical CRE provided a detailed description of the whole process from *bla*_KPC-2_ gene first occurrence, progress to final prevalence. We observed that the prevalence of KPC-2-producing *Enterobacterales* was mediated by multiple species and STs, and that *bla*_KPC-2_ gene was located on three diverse variants of Tn*1721* in multi-drug resistance (MDR) region of plasmid. IncFII was the most common Incompatibility group. Further study indicated that replicative transposition contributed to the evolution and dissemination of KPC-2-producing plasmid.

Transposable elements play an important role in the genetic variation and evolution of bacteria. The *bla*_KPC-2_ gene is mostly located on transposable elements, such as Tn*4401*, Tn*1721,* and IS*26* [8,13]. To date, eight variants of Tn*4401* (Tn*4401a* to Tn*4401h*) have been identified, with Tn*4401a* and Tn*4401b* being the most widespread [1,12,19,20]. Notably, these isoforms demonstrate lacking *tnpA* or/and *tnpR*, or have deletions in the noncoding region upstream of *bla*_KPC_ leading to enhanced or reduced expression of this carbapenemase [21]. It is widely reported that Tn*1721* variants are the dominant structures in Asia, particularly in China [8–10,15,22]. Given that the structure of Tn*1721* variants in current *bla*_KPC-2_ epidemic is too complicated and diverse to track the dissemination process of this gene, we focus on the early stage of *bla*_KPC-2_ epidemic. In addition to the dynamics of *bla*_KPC-2_-bearing transposon structure raised by this study, host and plasmid factors as well as antibiotic pressure from environment were also worth further study.

Our findings indicated that all three Tn*1721* variants were capable of mobilizing *bla*_KPC-2_ via replicative transposition, and A2- and B-type Tn*1721* exhibited higher transposition frequencies than A1-type. Such various capacities are presumably associated with cointegrate resolution of Tn*1721* in transposition assay. For the transconjugants with the Tn*1721* pattern, only the cointegrate could be conjugated into the recipient (*E. coli* HB101, STR resistant [STR^r^]) and screened on LB agar containing IPM, TMP, and STR in the transposition assay. Once the cointegrate resolved in donor strain, neither plasmid generated here could survive. Because P1 lacked the TMP resistance (TMP^r^) and the ability to conjugate into recipient (STR^r^), even though it possessed IPM resistance (IPM^r^), while P2 was an opposite case. In contrast, both the cointegrate and plasmid resolved from the cointegrate with Tn*1721*-*bla*_KPC-2_-IRL2 pattern possessed IPM^r^ and TMP^r^, as well as had the ability to conjugate to recipient (STR^r^). For the transposition via IS*26*, there is no resolution process. It means that the cointegrate (P3) with IS*26* pattern possessed IPM^r^ and TMP^r^, as well as had the ability of conjugal transfer. Consequently, it survived on LB agar containing IPM, TMP, and STR. In other words, the plasmid with Tn*1721*-*bla*_KPC-2_-IRL2 pattern (in B-type) or IS*26* pattern (in A2-type) had a certain superior ability of conjugal transfer and became more widespread. These results strongly supported our molecular epidemiological findings that the two peaks of *bla*_KPC-2_ epidemic followed the appearance of A2- and B-type Tn*1721*, reflecting an important role for replicative transposition of Tn*1721* and IS*26* in the evolution and transmission of *bla*_KPC-2_-carrying plasmid in *Enterobacterales*.

In conclusion, our work demonstrates replicative transposition facilitates the evolution and transmission of KPC-2-producing plasmid in *Enterobacterales*, and highlights its important role in the dissemination of antibiotic resistance genes between pathogenic bacterial species.

## Supplementary Material

Supplemental Material

Supplemental Material

## References

[1] Munoz-Price LS, Poirel L, Bonomo RA, et al. Clinical epidemiology of the global expansion of *Klebsiella pneumoniae* carbapenemases. Lancet Infect Dis. 2013;13(9):785–796. 23969216 10.1016/S1473-3099(13)70190-7 PMC4673667

[2] Mathers AJ, Peirano G, Pitout JD. The role of epidemic resistance plasmids and international high-risk clones in the spread of multidrug-resistant enterobacteriaceae. Clin Microbiol Rev. 2015;28(3):565–591. 25926236 10.1128/CMR.00116-14 PMC4405625

[3] Shen P, Zhang Y, Tang Y, et al. Molecular dissection of *bla*_KPC-2_-bearing plasmids evolving in *Klebsiella pneumoniae* isolated at one teaching hospital in shanghai, China. FEMS Microbiol Lett. 2016;363(15):fnw142. 27252157 10.1093/femsle/fnw142

[4] Peirano G, Bradford PA, Kazmierczak KM, et al. Importance of clonal complex 258 and IncF_K2-like_ Plasmids among a global collection of *Klebsiella pneumoniae* with *bla*KPC. Antimicrob Agents Chemother. 2017;61(4). 10.1128/AAC.02610-16 PMC5365689 28167556

[5] Naas T, Cuzon G, Villegas MV, et al. Genetic structures at the origin of acquisition of the beta-lactamase bla KPC gene. Antimicrob Agents Chemother. 2008;52(4):1257–1263. 18227185 10.1128/AAC.01451-07 PMC2292522

[6] Chen L, Chavda KD, Melano RG, et al. Complete sequence of a bla(KPC-2)-harboring IncFII(K1) plasmid from a *Klebsiella pneumoniae* sequence type 258 strain. Antimicrob Agents Chemother. 2013;57(3):1542–1545. 23295924 10.1128/AAC.02332-12 PMC3591897

[7] Pereira PS, de Araujo CF, Seki LM, et al. Update of the molecular epidemiology of KPC-2-producing *Klebsiella pneumoniae* in Brazil: spread of clonal complex11 (ST11, ST437 and ST340). J Antimicrob Chemother. 2013;68(2):312–316. 23070735 10.1093/jac/dks396

[8] Octavia S, Kalisvar M, Venkatachalam I, et al. *Klebsiella pneumoniae* and Klebsiella quasipneumoniae define the population structure of blaKPC-2Klebsiella: a 5 year retrospective genomic study in Singapore. J Antimicrob Chemother. 2019;74(11):3205–3210. 31504571 10.1093/jac/dkz332

[9] Fu P, Tang Y, Li G, et al. Pandemic spread of blaKPC-2 among *Klebsiella pneumoniae* ST11 in China is associated with horizontal transfer mediated by IncFII-like plasmids. Int J Antimicrob Agents. 2019;54(2):117–124. 30885806 10.1016/j.ijantimicag.2019.03.014

[10] Zhang W, Zhu Y, Wang C, et al. Characterization of a Multidrug-Resistant porcine *Klebsiella pneumoniae* sequence type 11 strain coharboring bla KPC-2 and fosA3 on Two Novel hybrid plasmids. mSphere. 2019 Sep 11;4(5). 10.1128/mSphere.00590-19 PMC6739495 31511369

[11] Woods LC, Gorrell RJ, Taylor F, et al. Horizontal gene transfer potentiates adaptation by reducing selective constraints on the spread of genetic variation. Proc Natl Acad Sci USA. 2020;117(43):26868–26875. 33055207 10.1073/pnas.2005331117 PMC7604491

[12] Cuzon G, Naas T, Nordmann P. Functional characterization of Tn4401, a Tn3-based transposon involved in blaKPC gene mobilization. Antimicrob Agents Chemother. 2011;55(11):5370–5373. 21844325 10.1128/AAC.05202-11 PMC3195030

[13] Tang Y, Li G, Liang W, et al. Translocation of carbapenemase gene blaKPC-2 both internal and external to transposons occurs via Novel structures of Tn1721 and exhibits distinct movement patterns. Antimicrob Agents Chemother. 2017;61(10). 10.1128/AAC.01151-17 PMC5610484 28784666

[14] Yang J, Ye L, Guo L, et al. A nosocomial outbreak of KPC-2-producing *Klebsiella pneumoniae* in a Chinese hospital: dissemination of ST11 and emergence of ST37, ST392 and ST395. Clin Microbiol Infect. 2013;19(11):E509–E515. 23841705 10.1111/1469-0691.12275

[15] Shen P, Zhang Y, Li G, et al. Characterization of the genetic environment of the blaKPC-2 gene among *Klebsiella pneumoniae* isolates from a Chinese hospital. Braz J Infect Dis. 2016;20(4):384–388. 27183358 10.1016/j.bjid.2016.04.003 PMC9427567

[16] Villa L, Garcia-Fernandez A, Fortini D, et al. Replicon sequence typing of IncF plasmids carrying virulence and resistance determinants. J Antimicrob Chemother. 2010;65(12):2518–2529. 20935300 10.1093/jac/dkq347

[17] Harmer CJ, Moran RA, Hall RM. Movement of IS26-associated antibiotic resistance genes occurs via a translocatable unit that includes a single IS26 and preferentially inserts adjacent to another IS26. mBio. 2014;5(5):e01801–14. 25293759 10.1128/mBio.01801-14 PMC4196232

[18] He S, Hickman AB, Varani AM, et al. Insertion sequence IS26 reorganizes plasmids in clinically isolated multidrug-resistant bacteria by replicative transposition. mBio. 2015 Jun 9;6(3):e00762. 26060276 10.1128/mBio.00762-15 PMC4471558

[19] Baraniak A, Izdebski R, Fiett J, et al. KPC-Like Carbapenemase-producing Enterobacteriaceae colonizing patients in Europe and Israel. Antimicrob Agents Chemother. 2016;60(3):1912–1917. 10.1128/AAC.02756-15 PMC4775924 26711772

[20] Pecora ND, Li N, Allard M, et al. Genomically informed surveillance for carbapenem-resistant Enterobacteriaceae in a health care system. mBio. 2015;6(4):e01030. 26220969 10.1128/mBio.01030-15 PMC4551976

[21] Cheruvanky A, Stoesser N, Sheppard AE, et al. Enhanced *Klebsiella pneumoniae* carbapenemase expression from a Novel Tn4401 deletion. Antimicrob Agents Chemother. 2017;61(6). 10.1128/AAC.00025-17 PMC5444142 28373185

[22] Chen YT, Lin JC, Fung CP, et al. KPC-2-encoding plasmids from *Escherichia coli* and *Klebsiella pneumoniae* in Taiwan. J Antimicrob Chemother. 2014;69(3):628–631. 24123430 10.1093/jac/dkt409

