# Supplementary material for: Replicative transposition contributes to the evolution and dissemination of KPC-2-producing plasmid in Enterobacterales
Source: Emerg Microbes Infect. 2021 Dec 21;11(1):113–22. doi: 10.1080/22221751.2021.2013105 (PMC8725868; doi:10.1080/22221751.2021.2013105)
Supplement: Supplemental Material [file TEMI_A_2013105_SM3604.docx]

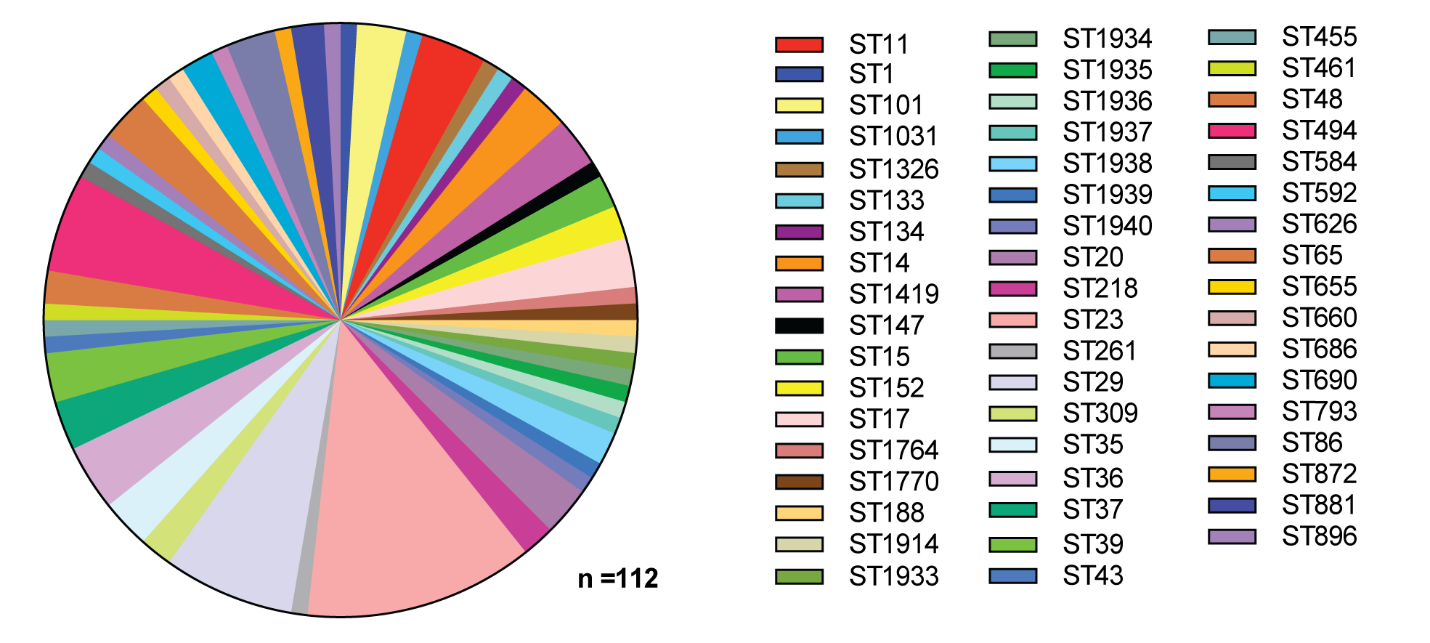


Figure S1. Multilocus sequence typing (MLST) distribution of CS-KP isolates.

Table S1. Primers used in this study.

| Primer | Sequence^a^ (5'-3') | Target |
| --- | --- | --- |
| JP283 | GGGAGCACATCAACCTAACC | Region internal to Tn*1721*-IRR |
| JP200 | ACCACCACTGTATCGCCTTC | Region internal to Tn*1721*-IRL |
| JP190 | CAACGTGAAGAAGTGGCAGA | Region internal to IS26-IRR |
| JP843 | CGTACGGCCCACAGAATGAT | Region internal to IS26-IRL |
| R388-F1 | CGACGCCAGAGACCGAG | R388 |
| R388-F2 | TTCCGCCGTCCAGATTAC | R388 |
| R388-F3 | GCAGATTGAAGGATGAAATTAG | R388 |
| R388-F4 | CTGCTTATGGTGCTGCTGTG | R388 |
| R388-F5 | CGGTATCACCCATAAGCACA | R388 |
| R388-F6 | AGCCAGACAAACAACACTCCA | R388 |
| R388-F7 | CTTCGTCAACTGTCATACCG | R388 |
| R388-F8 | GCATTGCTCTTGCTCCTG | R388 |
| R388-F9 | TTCCAAGGCTCCTGAGCA | R388 |
| R388-F10 | AAACGTCAGATTCGGTTAAAC | R388 |
| R388-F11 | GGCCGACCGTAAAGACTG | R388 |
| R388-F12 | TCGGTAACACCATGCTCATC | R388 |
| R388-F13 | TGGCTTGGAACGGGATT | R388 |
| R388-F14 | GGAATACCTCAATTCACTGAACA | R388 |
| R388-F15 | GTCCACCTCCCCACTCAAG | R388 |
| R388-F16 | GCTCATGCAGCAGTATCGTG | R388 |
| R388-F17 | CCCTCGACTAGAACCTCACG | R388 |
| R388-F18 | CACGTGTTCTGCCAACATAAC | R388 |
| R388-F19 | TGGATGGGAAGGCTTGAC | R388 |
| R388-F20 | TTGTGGGGTAGGCAGTCAG | R388 |
| R388-R1 | GAGGGTTTCCGAGAAGGTGATTG | R388 |
| R388-R2 | GACTTGTACCGAGATGTTGCTG | R388 |
| R388-R4 | AGTTGCCTCCTTTGTTGACG | R388 |
| R388-R5 | AGCAAACGCACGGAACAG | R388 |
| R388-R6 | TTGCTTGCTTTCCTTGACG | R388 |
| R388-R7 | GAGCAACAACAACGCGAAC | R388 |
| R388-R8 | CGAGCAGGAGAAAGACGAAC | R388 |
| R388-R9 | CCTGCCCTACGGTTCTATG | R388 |
| R388-R15 | TGTATCGGGCACCAACG | R388 |
| R388-R16 | TTTGTTGCTTTCTGCGCTTGG | R388 |
| R388-R17 | ACCGAACGACGAATGACGAG | R388 |
| R388-R20 | CGCATAGCCTTCAGGAGTG | R388 |
| JP934 | ATGTTGGATAGCAAGGACTATCAGTCCGGCAATGCTACATATC | IRR fragment forward |
| JP935 | TGGAAGACGGGAAGTTTCG | IRR fragment reverse |
| JP936 | CCGAAACTTCCCGTCTTCCATGTCAGTCGTCCGTG | tnpR fragment forward |
| JP937 | CAACGGAACAGAAAGTGC | tnpR fragment reverse |
| JP295 | TCGCTAAACTCGAACAGG | *bla*_KPC-2_-specific probe forward |
| JP296 | TTACTGCCCGTTGACGCCCAATCC | *bla*_KPC-2_-specific probe reverse |
| JP831 | CGGCGTGGGCTACCTGAAC | sul1-specific probe forward |
| JP832 | GAGGGTTTCCGAGAAGGTGATTG | sul1-specific probe reverse |
| JP282 | CGTGTTCCACAGCACGATAG | Tn1721-specific probe forward |
| JP1030 | TCAACCACCAGTATCGCCAC | Tn1721-specific probe reverse |
| TP24 | GGCGTTGATTATCCGTTTCAG | IS26-specific probe forward |
| TP25 | CATCATTCTGTGGGCCGTAC | IS26-specific probe reverse |
| TP26 | ATTCACCTGACGACGCAGC | lacY forward |
| TP27 | TGCCGCTATTTCTCTGTTCTCG | lacY reverse |

^a^ Underline indicates overlaps introduced by the primer.

Table S2. Plasmids used in this study

| Plasmid | Description | Resistance phenotype^a^ | Reference |
| --- | --- | --- | --- |
| R388^b^ | IncW plasmid harboring the sul1 gene | TMP | 17 |
| pHS10842^c^ | Clinical plasmid harboring the Tn1721- blaKPC-2-IS26 structure (A2-type Tn1721) | IPM | 13 |
| pHS10842-Tn*1721*A1 | pHS10842 derivative containing A1-type Tn1721 | IPM | 13 |
| pHS10842-Tn*1721*B | pHS10842 derivative containing B-type Tn1721 | IPM | 13 |
| pHS10842-ΔtnpA_Tn1721_ | Tn1721-blaKPC-2-IS26 with ΔtnpATn1721 deletion | IPM | This study |
| pHS10842-Δtnp26 | Tn1721-blaKPC-2-IS26 with Δtnp26 deletion | IPM | This study |
| pHS10842-ΔtnpA_Tn1721_Δtnp26 | Tn1721-blaKPC-2-IS26 with ΔtnpATn1721 and Δtnp26 deletion | IPM | This study |

^a^IPM, imipenem; TMP, trimethoprim.

^b^GenBank accession number BR000038.

^c^GenBank accession number KP125892.

Table S3. IncFII screen of clinical isolates with or without *bla*_KPC-2_.

| Isolate | *bla*_KPC-2_-positive^a^ | *bla*_KPC-2_-negative |
| --- | --- | --- |
| *K. pneumoniae* | 96/112 (85.71%) | 55/112 (49.11%) |
| *E. coli* | 11/15 (73.33%) | - |
| *Ctro. freundii* | 0/15 (0%) | - |
| *Ent. aerogenes* | 8/12 (66.67%) | - |
| Others | 2/7 (28.57%) | - |
| In total | 117/161(72.67%) | - |

^a^Data are number of IncFII-positive / total number (% of IncFII-positive rates). *P* value for comparisons of the IncFII-positive rates of *bla*_KPC-2_-positive and *bla*_KPC-2_-negative groups.
